# A novel accelerometer-based method to describe day-to-day exposure to potentially osteogenic vertical impacts in older adults: findings from a multi-cohort study

**DOI:** 10.1007/s00198-016-3810-5

**Published:** 2016-10-31

**Authors:** K. Hannam, K. C. Deere, A. Hartley, E. M. Clark, J. Coulson, A. Ireland, C. Moss, M. H. Edwards, E. Dennison, T. Gaysin, R. Cooper, A. Wong, J. S. McPhee, C. Cooper, D. Kuh, J. H. Tobias

**Affiliations:** 1 Musculoskeletal Research Unit, University of Bristol School of Clinical Sciences, Bristol, BS10 5NB UK; 2 School of Healthcare Sciences, Manchester Metropolitan University, Manchester, M15 6BH UK; 3 MRC Lifecourse Epidemiology Unit, University of Southampton, Southampton, SO16 6YD UK; 4 MRC Unit for Lifelong Health and Ageing at UCL, London, WC1E 6BT UK

**Keywords:** Accelerometry, Bone, Older adults, Physical activity

## Abstract

**Summary:**

This observational study assessed vertical impacts experienced in older adults as part of their day-to-day physical activity using accelerometry and questionnaire data. Population-based older adults experienced very limited high-impact activity. The accelerometry method utilised appeared to be valid based on comparisons between different cohorts and with self-reported activity.

**Introduction:**

We aimed to validate a novel method for evaluating day-to-day higher impact weight-bearing physical activity (PA) in older adults, thought to be important in protecting against osteoporosis, by comparing results between four cohorts varying in age and activity levels, and with self-reported PA levels.

**Methods:**

Participants were from three population-based cohorts, MRC National Survey of Health and Development (NSHD), Hertfordshire Cohort Study (HCS) and Cohort for Skeletal Health in Bristol and Avon (COSHIBA), and the Master Athlete Cohort (MAC). *Y*-axis peaks (reflecting the vertical when an individual is upright) from a triaxial accelerometer (sampling frequency 50 Hz, range 0–16 g) worn at the waist for 7 days were classified as low (0.5–1.0 g), medium (1.0–1.5 g) or higher (≥1.5 g) impacts.

**Results:**

There were a median of 90, 41 and 39 higher impacts/week in NSHD (age 69.5), COSHIBA (age 76.8) and HCS (age 78.5) participants, respectively (total *n* = 1512). In contrast, MAC participants (age 68.5) had a median of 14,322 higher impacts/week. In the three population cohorts combined, based on comparison of beta coefficients, moderate-high-impact activities as assessed by PA questionnaire were suggestive of stronger association with higher impacts from accelerometers (0.25 [0.17, 0.34]), compared with medium (0.18 [0.09, 0.27]) and low impacts (0.13 [0.07,0.19]) (beta coefficient, with 95 % CI). Likewise in MAC, reported moderate-high-impact activities showed a stronger association with higher impacts (0.26 [0.14, 0.37]), compared with medium (0.14 [0.05, 0.22]) and low impacts (0.03 [−0.02, 0.08]).

**Conclusions:**

Our new accelerometer method appears to provide valid measures of higher vertical impacts in older adults. Results obtained from the three population-based cohorts indicate that older adults generally experience very limited higher impact weight-bearing PA.

**Electronic supplementary material:**

The online version of this article (doi:10.1007/s00198-016-3810-5) contains supplementary material, which is available to authorized users.

## Introduction

Hip fracture is a major cause of morbidity and mortality in older people, leading to loss of independence, and a huge economic burden through both direct medical costs and social sequelae [1]. It is thought that age-related declines in the intensity and quantity of physical activity (PA) contribute to this increase in risk of osteoporotic fracture. Promotion of PA in older people is thought to help maintain bone mass: epidemiological studies report that risk of hip fracture is reduced in older adults who remain more active [2]. There is little evidence that walking interventions improve bone mineral density (BMD), as judged by findings of a 2008 meta-analysis [3]. In contrast, protocols that combined jogging, walking and stair climbing consistently improve hip BMD in older people [4]. Interventions to increase aerobic activities, high-impact exercises, “odd-impact” exercise loading and resistance training (designed to increase bone loading through increased muscle strength) also improve hip BMD in this age group [4–8]. These findings are consistent with those from laboratory studies conducted over 30 years ago, demonstrating that dynamic mechanical loading exerts a dose-response effect on bone formation [9].

Whereas several studies in older individuals suggest that high impacts are beneficial for the bone, interventional trials are generally of short duration. Epidemiological studies have examined the benefits of habitual participation in high-impact PA for skeletal health, based on use of questionnaires asking about day-to-day participation in different activities. For example, lifetime sport and leisure participation was found to be positively related to hip size and strength in older men, using an osteogenic index to score different activities according to impact level [10]. One of the limitations of PA questionnaires is that they do not provide an objective measurement of PA, and may fail to include brief or non-volitional, but nonetheless important periods of activity. Accelerometry provides an alternative means of evaluating habitual levels of PA, which overcomes these limitations. However, accelerometry studies based on widely used actigraph devices generally classify intensity using counts per minute thresholds calibrated against energy expenditure, which may not be ideally suited for analysing relationships with bone outcomes. In addition, in deriving counts per minute, movement frequency and acceleration magnitude are combined using proprietary software, making it impossible to evaluate PA according to level of impact. For example, in a recent study of 1228 men and women aged 70 years, habitual levels of PA as assessed by accelerometry were positively related to hip BMD, but similar relationships were observed for activity of both moderate and vigorous intensity based on conventional counts per minute cut-points, suggesting these approaches are unable to identify specific osteogenic components of day-to-day weight-bearing PA [11].

Alternative accelerometer outputs may be more suitable for evaluating relationships between day-to-day PA and skeletal health. For example, the Newtest device was designed to start sampling vertical accelerations each time these exceed a threshold of 0.3 g, following which the maximum acceleration value was identified and recorded, representing the acceleration peak for a given movement [12]. Positive relationships between PA participation and hip BMD could be fully explained by the number of impacts above 4 g as measured in this way [13, 14]. As well as the latter studies performed in adolescents and premenopausal women, exercise training leading to osteogenic impacts >3.9 g was found to have beneficial effects for femoral neck BMD in postmenopausal women between aged 50–66 years of age [15]. However, despite the potential importance of high-impact PA for skeletal health, we are not aware of previous attempts to characterise these levels using objective accelerometer-based methods in older adults who are at higher risk of osteoporosis.

Improvements in technology have made it possible to store an entire week of continuous accelerometer recordings, enabling acceleration peaks to be identified retrospectively by applying data processing algorithms to raw data [16]. In addition, since acceleration peaks are recorded in time sequence, artefacts and errors can be identified more readily [16]. In the present study, we aimed to use this approach to characterise day-to-day exposure to higher vertical impacts in older individuals, based on four different cohorts of which three were population based, and one recruited on the basis of high PA participation (Master Athlete cohort (MAC)). We also aimed to confirm the validity of this method by examining face validity as judged by whether our method could detect the relatively high levels of high-impact PA in MAC. Furthermore, we aimed to evaluate convergent validity based on relationships between PA as assessed using our novel accelerometry method and questionnaire.

## Methods

### Study populations

Four older adult UK cohorts contributed to the VIBE study, collecting questionnaire and accelerometry data between 2013 and 2016: the MRC National Survey of Health and Development (NSHD), Hertfordshire Cohort Study (HCS), Cohort for Skeletal Health in Bristol and Avon (COSHIBA) and the Master Athlete Cohort (MAC). NSHD and HCS are both well-established birth cohorts with extensive longitudinal data which have been used to explore the relationships of early life factors with health in later life. NSHD is a nationally representative sample of 5362 legitimate single births from 1 week in March 1946 [17, 18]. All participants included in the most recent NSHD data collection phase (2015–2016) were invited to participate in VIBE. HCS comprises singleton men and women who were born in Hertfordshire between 1931 and 1939 and still lived in the area during 1998–2003 [19]. In the HCS cohort, only participants who were previously included in the UK arm of the European Project on Osteoarthritis (EPOSA) [20] were invited to participate in VIBE. COSHIBA was a representative population-based cohort of 3200 women recruited through 15 general practices in the Bristol and Avon area during 2007–2009, originally set up to investigate determinants of skeletal health in postmenopausal women [21]. Only the 1280 COSHIBA participants who consented to be contacted about future research studies in 2014 and remained resident in the Bristol and Avon area were eligible to participate in the VIBE study. The MAC was set up de novo for the purpose of the VIBE study and recruited UK-based male and female master athletes who had competed in sprint, middle or long distance running in the past 12 months at a regional level at the time of recruitment. Separate regional ethical approval was obtained for VIBE study data collection in NSHD (14/LO/1073 and 14/SS/1009), HCS (10/HO311/59), COSHIBA (14/SW/0138) and MAC (14/NW0275), and written informed consent was obtained from all participants.

### Objective PA data—accelerometry

Participants who were invited and agreed to accelerometry monitoring, subject to availability of monitors, were provided with a GCDC X15-1c triaxial accelerometer (Gulf Coast Data Concepts, Waveland, Mississippi), custom designed size specific elasticated belt, a time log and a stamped addressed package along with written and, if seen in clinic, verbal instructions. Accelerometers were configured with standardised settings prior to participant use with a sampling frequency of 50 Hz, a deadband setting of 0.1 g (the threshold which must be exceeded before a recording is made) and a timeout setting of 10 s (a single sample every 10 s is forced even if the recording is <0.1 g). Participants were instructed (with a demonstration and/or instructions) to wear the accelerometer securely positioned in the belt over their right hip pointing toward the centre of their body for seven continuous days, removing only for sleeping, washing and swimming. A time log was provided for participants to record when the monitor was put on in the morning and taken off at night for each monitoring day and to state if there was any reason why that day had not been reflective of their normal activity. Raw triaxial accelerometry data were uploaded to a secure shared drive and read into Stata 13 (StataCorp, College Station, TX) for standardised cleaning and processing by the coordinating centre, described in detail elsewhere [16]. In short, *Y*-axis accelerations data were cleaned to remove movement artefacts and non-wear time. Activity data were normalised based on seven valid days (≥10 h recording time) of 14 h. *Y*-axis peaks were calculated based on accelerations higher than the preceding and subsequent reading and recorded within 14 pre-specified g bands. These were condensed to three distinct impact bands to reflect low (≥0.5 to <1.0 g), medium (≥1.0 to <1.5 g) and higher (≥1.5 g) impact. All g values represent g over and above 1 g from earth’s gravitational force.

### Self-reported questionnaire data

Participants completed a questionnaire collecting demographic, lifestyle and PA information at the time accelerometer monitoring began. Reported date of birth used to calculate participant age and height and weight for body mass index calculations were cross checked with previous cohort records for consistency. Self-rated health was reported from very good to very poor (participants from NSHD reported excellent to poor), and objective functional status was ascertained from short physical performance battery (SPPB) scores where available; SPPB data was collected at the time or shortly prior (approximately 1 month) from when the questionnaire was completed in MAC and COSHIBA and HCS data was taken from a 2010–2011 data collection. The main occupation during working life from each participant and their spouse (if married) were assigned a SOC90 (Standard Occupation Classification) code to obtain a proxy measure of social class. The highest SOC90 code between the participant and spouse was assigned where both occupations were provided and the codes were subsequently collapsed into four groups.

The PA questionnaire collected data on self-reported walking speed, number of flights of stairs climbed per day, active transport, past walking and weight-bearing PA across life and current PA activities with specific information about type and duration during the past 7 days. Duration was reported as approximate hours by indicating one of four categories: ‘Less than an hour’, ‘1-2 hours’, ‘2-4 hours’ and ‘more than 4 hours’. Participants were also asked if the PA they had recalled from the past 7 days were normal as compared to the rest of the year. The reported specific activities were then categorised into non-impact, low, moderate or high impact based on previous studies categorising activities based on ground reaction forces (GRF) (low: <2 times body weight (BW), moderate: 2–4 times BW, high: >4 times BW) [10] (see supplementary Table 1). Reported activities which had not been previously categorised were matched to the most similar categorised activity with known GRF data and assigned to that category (e.g. Yoga was categorised as low-impact based on a match to Tai Chi). All included activities assigned to each impact category are listed in supplementary Table 1. The high-impact category was merged with the moderate category as few participants reported engaging in the one activity which was categorised as high impact (i.e. sprint training).

### Statistical analysis

Participant demographic data and PA questionnaire responses were presented using means and standard deviations for continuous variables and the number and percentages for categorical variables. Due to the positively skewed nature of the accelerometry data, the median and 25th/75th percentiles of the absolute values were presented for each cohort stratified by gender. The accelerometry data for all other analyses were log transformed. *T* tests were used to explore gender differences between log accelerometry count data. To explore relationships between accelerometry data and self-reported PA questionnaire variables, univariate linear regression analyses were performed between PA questionnaire variables and *Y*-axis accelerometry peaks within low, medium and high bands. PA questionnaire variables comprised miles walked each day since the age of 50, flights of stairs climbed in a typical day, walking speed and approximate hours spent performing activities categorised as non, low and medium-high impact. Sensitivity analyses were performed restricted to those participants with at least three valid accelerometry days. Analyses were performed for each cohort individually and with the population-based cohorts (NSHD, COSHIBA and HCS) pooled.

## Results

**Fig. 1:**
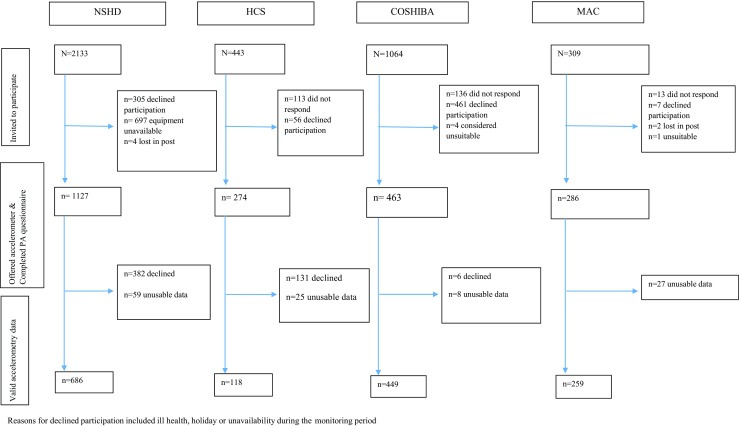
Recruitment flow diagram

Of the 2307 participants from the four cohorts who were invited to wear an accelerometer, a total of 1512 had valid accelerometry data in combination with questionnaire data; 686 were participants from NSHD, 449 from COSHIBA, 259 from MAC and 118 from HCS (Fig. 1). Only participants with both valid accelerometry and questionnaire data were included in the analyses presented.

### Participant characteristics

Overall, participants had a mean age of 72 (SD 5), with MAC and NSHD including the youngest participants (mean 69.5, SD 0.2), and HCS the oldest (mean 78.5, SD 2.6) (Table 1). COSHIBA was all-female, whereas NSHD, MAC and HCS comprised 51, 77 and 61 %, males respectively. Self-rated health was predominantly excellent to good (86 %) and this was reflected in high average SPPB scores. MAC participants had considerably higher reported health status (98 % very good or good) and a mean SPPB score of the maximum score of 12. Over half (53 %) of participants’ main lifetime occupation were in the top two SOC90 groups indicative of a high occupational class. MAC participants reported more walking and weight-bearing activity since the age of 50 compared to the three population-based cohorts, faster walking speed, and considerably more moderate-high-impact activity (Table 2). The latter finding reflected high rates of participation in running/jogging, as expected due to the selection criteria (see supplementary Table 2).

**Table 1:** Demographic data from all VIBE cohorts with questionnaire and valid accelerometry data

	NSHD	COSHIBA	MAC	HCS	Total
Total (*n* (% of total VIBE sample))	686 (45.37)	449 (29.70)	259 (17.13)	118 (7.80)	1512
Gender (*n* (%) female)	336 (48.98)	449 (100)	59 (22.87)	46 (38.98)	890 (58.9)
Age (mean (SD))	69.52 (0.22)	76.80 (3.08)	68.50 (6.25)	78.49 (2.62)	71.97 (5.13)
BMI kg/m^2^ (mean (SD))	27.16 (4.02)	26.81 (4.67)	22.27 (2.66)	25.87 (3.56)	26.09 (4.38)
Self-rated health (*n* (%))					
Excellent	66 (10.20)	–	–	–	66 (4.52)
Very good	301 (46.52)	110 (24.83)	174 (67.70)	16 (14.16)	601 (41.16)
Good	202 (31.22)	243 (54.85)	78 (30.35)	64 (56.64)	587 (40.21)
Fair	72 (11.13)	82 (18.51)	5 (1.95)	28 (24.78)	187 (12.81)
Poor	6 (0.93)	8 (1.81)	0 (0.00)	5 (4.42)	19 (1.30)
Very poor	–	0 (0.00)	0 (0.00)	0 (0.00)	0 (0.00)
Occupational class (SOC 90) (*n* (%)) (highest)^b^					
Class groups 1–2	346 (53.16)	211 (50.24)	170 (66.41)	37 (32.74)	764 (53.06)
Class groups 3–5	234 (35.94)	167 (39.76)	59 (23.05)	54 (47.79)	514 (35.69)
Class groups 6–8	66 (10.14)	40 (9.52)	23 (8.98)	20 (17.70)	149 (10.35)
Other occupations	5 (0.77)	2 (0.48)	4 (1.56)	2 (1.77)	13 (0.90)
Short physical performance battery score (mean(SD))^a^	Not available	9.7 (2.5)	12.0 (0.2)	8.9 (2.3)	

SOC90 groups 1–2: managers/administrators and professional; groups 3–5: associate professional and technical, clerical and secretarial and craft and related occupations; groups 6–8: personal and protective services, sales and plant and machine operatives.

^a^SPPB score for HCS taken from 2010–2011 data collection

^b^Highest SOC 90 code taken if a married couple

**Table 2:** Physical activity questionnaire descriptives

Questionnaire variables	NSHD	COSHIBA	MAC	HCS	Total
	*N* = 686	*N* = 449	*N* = 259	*N* = 118	*N* = 1512
Walking/cycling most days (*n* (%))					
No	220 (32.74)	151 (34.16)	72 (27.91)	30 (25.42)	473 (31.74)
Walk	416 (61.90)	277 (62.67)	130 (50.39)	81 (68.64)	904 (60.67)
Cycle	4 (0.60)	0 (0.00)	4 (1.55)	0 (0.00)	8 (0.54)
Both	32 (4.76)	14 (3.17)	52 (20.16)	7 (5.93)	105 (7.05)
No. of flight of stairs in a typical day (*n* (%))					
None	95 (13.99)	62 (13.90)	17 (6.59)	13 (11.02)	187 (12.46)
1–2	39 (5.74)	24 (5.38)	14 (5.43)	10 (8.47)	87 (5.80)
3–4	82 (12.08)	76 (17.04)	20 (7.75)	19 (16.10)	197 (13.12)
5–10	275 (40.50)	172 (38.57)	84 (32.56)	49 (41.53)	580 (38.64)
>10	188 (27.69)	112 (25.11)	123 (47.67)	27 (22.88)	450 (29.98)
Self-reported walking speed (*n* (%))					
Unable to walk	1 (0.15)	1 (0.22)	0 (0.00)	0 (0.00)	2 (0.13)
Very slow	17 (2.50)	41 (9.17)	0 (0.00)	13 (11.02)	71 (4.73)
Stroll at easy pace	97 (14.29)	105 (23.49)	15 (5.81)	41 (34.75)	258 (17.18)
Normal speed	358 (52.72)	193 (43.18)	73 (28.29)	49 (41.53)	673 (44.81)
Fairly brisk	187 (27.54)	99 (22.15)	139 (53.88)	10 (8.47)	435 (28.96)
Fast	19 (2.80)	8 (1.79)	31 (12.02)	5 (4.24)	63 (4.19)
Miles walked each day (since age 50) (*n* (%))					
<1 mile	162 (24.47)	111 (26.12)	28 (11.11)	32 (27.83)	333 (22.90)
1–2 miles	284 (42.90)	211 (49.65)	106 (42.06)	53 (46.09)	654 (44.98)
3–5 miles	160 (24.17)	83 (19.53)	74 (29.37)	21 (18.26)	338 (23.25)
>5 miles	56 (8.46)	20 (4.71)	44 (17.46)	9 (7.83)	129 (8.87)
Weight-bearing activity (since age 50) (*n* (%))					
None	378 (59.25)	159 (39.95)	1 (0.39)	58 (55.77)	596 (42.66)
Once a month	99 (15.52)	72 (18.09)	3 (1.17)	17 (16.35)	191 (13.67)
Once a week	87 (13.64)	99 (24.87)	10 (3.89)	16 (15.38)	212 (15.18)
More than once a week	74 (11.60)	68 (17.09)	243 (94.55)	13 (12.50)	398 (28.49)
Activities in the past 7 days (approx. hours mean(SD))					
Non-impact	1.57 (1.81)	1.37 (1.55)	2.04 (2.05)	1.40 (1.43)	1.58 (1.78)
Low-impact	5.69 (3.87)	4.98 (3.38)	6.58 (3.99)	5.56 (4.00)	5.62 (3.80)
Moderate-high impact	0.32 (0.89)	0.56 (1.16)	3.61 (1.83)	0.25 (0.93)	0.95 (1.70)

Compared to those invited but who did not participate, COSHIBA participants were younger whereas HCS participants were of similar age (supplementary Table 3). When compared with the remainder of the cohort, NSHD, COSHIBA and HCS participants with accelerometry data had a lower BMI and were more likely to have higher educational attainment.

### Accelerometer descriptives

Data from 119 worn and returned accelerometers were deemed unusable due to insufficient or corrupt data (Fig. 1). The average valid accelerometer wear time (based on ≥ 10 h recording for any given day) across all cohorts was 5.1 (SD 2.0) days (COSHIBA 5.2 (SD 1.8), MAC 6.0 (SD 1.6), HCS 4.2 (SD 2.2) and NSHD 4.9 (SD 2.1)). At least 3 valid days were captured for 93 % of participants. Sensitivity analyses were performed restricted to those participants with at least three valid days and similar results were observed; thus, results with all available valid accelerometry data are presented (normalised to 7 days of 14 h).

**Fig. 2:**
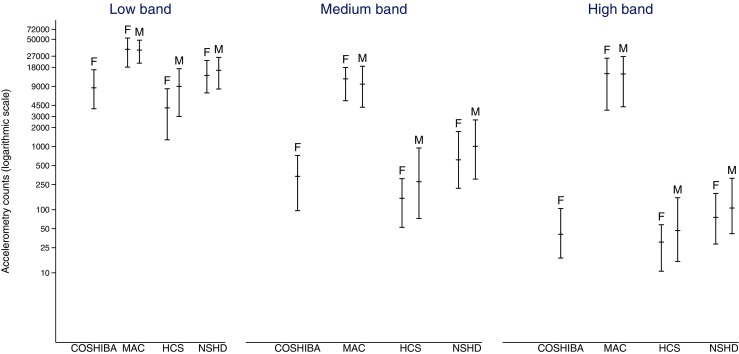
Figure shows accelerometry counts normalised to 7 days wear time in low (0.5 ≤ g < 1.0 g), medium (1.0 ≤ g < 1.5) and higher (≥1.5 g) bands (g units over and above 1 g of earth’s gravitational force) separated by sex. *M* male, *F* female. *Bars* represent the median, 25th percentile and 75th percentile of accelerometry counts

All cohorts experienced the greatest number of counts in the low accelerometry band (Fig. 2). For all bands, HCS experienced the fewest counts, followed by COSHIBA, NSHD and then MAC. The population-based cohorts experienced relatively few counts in the higher band (NSHD: 90, COSHIBA: 41 and HCS: 39, median counts per week), for which differences compared to MAC were particularly striking (MAC: 14,322). Males and females in MAC experienced similar number of counts in all three bands. In contrast, males generally had higher PA levels compared to females in the population-based cohorts. In HCS, males experienced more counts than females in the low (males 8978, females 4107 *p* = 0.01) and medium (males 278, females 152 *p* = 0.03) bands. In NSHD, more counts were experienced by the males in the medium (males 1012, females 618 *p* = 0.01) and higher bands (males 106, females 76 *p* = 0.01).

### Relationships between accelerometry and PA questionnaires

Subsequently, we examined results of linear regression analysis between PA questionnaire responses and accelerometry in data pooled from the three population-based cohorts. The results of the pooled analyses were almost unchanged following adjustment for cohort; thus, the unadjusted results are presented. Similar positive associations were seen between miles walked each day since age 50, number of flights of stairs climbed in a typical day, self-reported walking speed, and participation in non- and low-impact activities in the past 7 days, and low, medium and high impacts (Table 3). In contrast, based on comparison of beta coefficients, there was a suggestion that reported moderate-high-impact activities showed stronger associations with higher impacts (0.25 [0.17, 0.34]), compared with medium (0.18 [0.09, 0.27]) and low impacts (0.13 [0.07, 0.19]) (Table 3) (beta coefficients, with 95 % CI). Similar associations were observed in analyses based on specific cohorts (supplementary Tables 4, 5 and 6 for COSHIBA, HCS and NSHD respectively), with the exception that in HCS, miles walked each day since age 50 were preferentially related to low impacts.

**Table 3:** Linear regression analyses exploring associations between physical activity questionnaire data and accelerometry in three population-based cohorts (COSHIBA, HCS and NSHD)

Questionnaire variable	Low accelerometry band				Medium accelerometry band				High accelerometry band			
	*N*	Median	Beta (95 % CI)	*p*	*N*	Median	Beta (95 % CI)	*p*	*N*	Median	Beta (95 % CI)	*p*
Miles walked each day (since age 50)	1161	11,536.19	0.39 (0.32, 0.46)	<0.01	1161	486.31	0.46 (0.36, 0.56)	<0.01	1161	62.27	0.37 (0.26, 0.47)	<0.01
<1 mile	289	6877.07			289	253.33			289	39.83		
1–2 miles	533	11,443.90			533	514.09			533	68.78		
3–5 miles	255	15,343.65			255	737.28			255	87.70		
>5 miles	84	22,472.70			84	872.68			84	91.93		
No. of flight of stairs in a typical day	1198	11,368.89	0.16 (0.12, 0.21)	<0.01	1198	482.87	0.22 (0.15, 0.29)	<0.01	1198	61.64	0.17 (0.10, 0.23)	<0.01
None	160	8975.19			160	233.92			160	42.33		
1–2	72	10,733.72			72	387.23			72	66.31		
3–4	166	8472.46			166	285.87			166	42.78		
5–10	479	11,945.42			479	486.31			479	63.80		
>10	321	13,277.53			321	680.60			321	99.13		
Self-reported walking speed	1199	11,348.74	0.62 (0.55, 0.68)	<0.01	1199	481.22	0.79 (0.70, 0.89)	<0.01	1199	61.75	0.59 (0.49, 0.68)	<0.01
Unable to walk	1	746.80			1	22.60			1	6.78		
Very slow	63	1908.36			63	78.79			63	20.26		
Stroll at easy pace	233	6073.78			233	177.38			233	32.11		
Normal speed	582	11,868.43			582	527.99			582	62.84		
Fairly brisk	289	19,195.57			289	1045.07			289	113.61		
Fast	31	23,622.97			31	1064.14			31	98.89		
Activities in the past 7 days (approx hours)^a^												
Non-impact			0.10 (0.06, 0.14)	<0.01			0.13 (0.07, 0.18)	<0.01			0.10 (0.05, 0.15)	<0.01
Low-impact			0.09 (0.08, 0.11)	<0.01			0.12 (0.10, 0.14)	<0.01			0.10 (0.07, 0.12)	<0.01
Moderate-high impact			0.13 (0.07, 0.19)	<0.01			0.18 (0.09, 0.27)	<0.01			0.25 (0.17, 0.34)	<0.01

Table shows associations between number of low (≥0.5 g to <1.0 g), medium (≥1.0 g to <1.5 g) and high (≥1.5 g) impacts normalised to 7 days and self-reported PA in the pooled population-based cohorts. Median represents median number of accelerometry counts within the low, medium and high accelerometry band for each questionnaire category. Beta coefficients represent change in log accelerometry counts across PA questionnaire categories (and change in log accelerometry counts per hour of reported activity).

^a^Self-reported activities categorised by impact level presented in supplementary Table 1

Broadly similar results were observed in MAC where self-reported walking speed, and participation in non- and low-impact activities showed equivalent positive associations with low, medium and higher impacts (Table 4). As in the population-based cohorts, reported moderate-high-impact activities showed a stronger association with higher impacts (0.26 [0.14, 0.37]), compared with medium (0.14 [0.05, 0.22]) and low impacts (0.03 [−0.02, 0.08]) (Table 4). In addition, evidence that these associations differed was stronger than for population-based cohorts, since confidence intervals for beta coefficients of regressions for self-reported moderate-high and low impacts were non-overlapping. Although miles walked each day since age 50 were related to all impact bands, in contrast to the population-based cohorts, stronger associations were observed for higher (0.40 [0.16, 0.64]) as compared with medium (0.28 [0.11, 0.45]) or low impacts (0.13 [0.03, 0.23]). In addition, no association was observed between number of flights of stairs climbed in a typical day and any impacts.

**Table 4:** Linear regression analyses exploring associations between physical activity questionnaire data and accelerometry in the Master Athlete cohort

Questionnaire variable	Low accelerometry band				Medium accelerometry band				High accelerometry band			
	*N*	Median	Beta (95 % CI)	*p*	*N*	Median	Beta (95 % CI)	*p*	*N*	Median	Beta (95 % CI)	*p*
Miles walked each day (since age 50)	249	33,946.79	0.13 (0.03, 0.23)	0.01	249	10,654.82	0.28 (0.11, 0.45)	<0.01	249	14,394.34	0.40 (0.16, 0.64)	<0.01
<1 mile	28	22,795.83			28	7279.81			28	5588.09		
1–2 miles	104	32,193.47			104	9281.18			104	13,550.93		
3–5 miles	73	37,050.40			73	10,664.37			73	16,050.86		
>5 miles	44	36,694.82			44	15,093.36			44	17,881.44		
No. of flight of stairs in a typical day	255	33,796.14	0.03 (−0.05, 0.10)	0.49	255	10,064.36	0.06 (−0.08, 0.19)	0.41	255	14,344.67	0.13 (−0.05, 0.32)	0.16
None	17	28,748.02			17	6233.90			17	8380.68		
1–2	14	30,226.39			14	8593.43			14	13,289.91		
3–4	20	30,375.67			20	6623.55			20	7627.13		
5–10	82	34,534.63			82	10,136.88			82	16,348.45		
>10	122	34,355.14			122	10,782.99			122	15,069.09		
Self-reported walking speed	255	33,796.14	0.23 (0.11, 0.34)	<0.01	255	10,064.36	0.39 (0.19, 0.60)	<0.01	255	14,344.67	0.29 (−0.01, 0.58)	0.06
Unable to walk	0	0.00			0	0.00			0	0.00		
Very slow	0	0.00			0	0.00			0	0.00		
Stroll at easy pace	14	30,551.51			14	6847.65			14	22,518.78		
Normal speed	71	31,585.13			71	7879.40			71	8074.59		
Fairly brisk	139	33,445.60			139	9989.79			139	14,918.62		
Fast	31	44,347.29			31	20,690.11			31	17,070.57		
Activities in the past 7 days (approx hours)^a^												
Non-impact			0.04 (−0.01, 0.08)	0.11			0.04 (−0.04, 0.11)	0.31			0.03 (−0.08, 0.13)	0.61
Low-impact			0.02 (0.00, 0.04)	0.07			0.02 (−0.02, 0.06)	0.36			-0.00 (−0.06, 0.05)	0.98
Moderate-high impact			0.03 (−0.02, 0.08)	0.28			0.14 (0.05, 0.22)	<0.01			0.26 (0.14, 0.37)	<0.01

Table shows associations between number of low (≥0.5 to <1.0 g), medium (≥1.0 to <1.5 g) and high (≥1.5 g) impacts normalised to 7 days and self-reported PA in the MAC. Median represents median number of accelerometry counts within the low, medium and high accelerometry band for each questionnaire category. Beta coefficients represent change in log accelerometry counts across PA questionnaire categories (and change in log accelerometry counts per hour of reported activity).

^a^Self-reported activities categorised by impact level presented in supplementary Table 1

## Discussion

We used our recently developed accelerometer-based method to evaluate day-to-day exposure to higher vertical impacts in older individuals. In all three population-based cohorts examined, very few higher impacts were observed. For example in HCS, which was our oldest cohort, individuals performed a median of only five higher impacts daily. Gender differences in high-impact PA, which we previously observed in adolescents [13], appeared to persist into older age, with approximately 50 % more higher impacts observed in male as compared with female participants in both NSHD and HCS. Nonetheless, although male NSHD participants experienced relatively large numbers of higher impacts when compared with the two other population-based cohorts, this still only equated to 15 higher impacts per day. These findings are consistent with previous observations that older adults partake in relatively little intense activity [22, 23]. For example, in the recent study by Johansson et al., 70 year old women only undertook a mean of 3 min vigorous PA daily, based on a conventional metabolic rate threshold [11]. The present study suggests that this limited level of exposure applies equally if not more so to higher impact PA.

Our findings support the validity of our method for measuring habitual levels of higher impact weight-bearing PA. For example, this appears to have face validity, based on our observation of considerably more higher vertical impacts in MAC as compared with population-based cohorts, which is expected given the former was recruited on the basis of high levels of habitual weight-bearing PA. Similarly, differences in the amount of higher impact PA between population cohorts were in line with age differences, with the highest levels observed in the youngest cohort (i.e. NSHD), and lowest levels in the oldest cohort (i.e. HCS). There was also evidence that our accelerometry method has convergent validity, since higher impacts as determined by accelerometry were preferentially related to participation in moderate-high, compared with low or medium, impact activities, as assessed by questionnaire. That said, the strength of this association (standardised beta coefficient approximately 0.25) was slightly weaker than that seen in previous studies comparing indirect and direct measures of PA intensity in older adults (*r* = 0.35 to 0.39) [24, 25]. As well as reflecting inaccuracies in self-reported higher impact PA, relatively weak relationships between accelerometer- and questionnaire-based measures of PA may be explained by the fact that accelerometer readings also reflect the intensity with which any given activity is carried out. For example, in our recent study of older individuals attending an aerobics classes, a given activity produced a wide range of peak accelerations [26]. In contrast, self-reported gait speed showed relatively strong associations with accelerometry recordings, across all bands, suggesting that intensity with which PA is undertaken may be a more important determinant of exposure to high vertical impacts than its frequency.

The ability to accurately record day-to-day exposure to high vertical impacts using accelerometers will enable new insights to be gained as to how habitual PA influences skeletal health. Understanding this is important in designing PA interventions intended to reduce adverse consequences, such as hip fracture. As discussed above, this rationale is based on previous studies of adolescents and premenopausal women using Newtest accelerometers, which suggest that relationships between PA and hip BMD are solely explained by exposure to high vertical impact [13, 14]. However, whereas the latter studies used 4 g to denote high impacts, it was not feasible to apply this threshold in the present study since accelerations of this magnitude were not observed in free living unselected older individuals. Instead, we used a 1.5-g threshold to define ‘higher’ impacts, based on the frequency of impacts in different bands observed in our previous pilot study; in particular, virtually no impacts were seen beyond 2 g in a sub-sample of HCS participants [16]. Although considerably lower than the 4-g threshold used in younger individuals, 1.5 g appears to represent a reasonable target in terms of high impacts in older individuals. Medium-impact activities such as walking do not generally reach 1.5 g, but this threshold is achieved in several components of aerobics exercise classes performed by older adults [26].

Although use of a 1.5-g threshold represents a pragmatic approach to defining higher vertical impacts in older individuals, how useful this is in defining bone protective activity is currently unclear and can only be determined by further studies where these are related to bone outcomes. In fact, on relating accelerometry data from COSHIBA to bone outcomes collected concurrently, we recently observed that higher impacts as defined using the 1.5-g threshold are positively related to lower limb bone strength, as reflected by hip cross sectional moment of inertia as measured by dual energy X-ray absorptiometry (DXA), in contrast to medium or low impacts (K Hannam et al., submitted for publication). Therefore, in spite of their rarity and the low g level used to define them, day-to-day higher vertical impacts in older individuals also appear to have specific bone protective properties as found in younger individuals, underscoring the importance of validated methods for recording them, as reported here.

While the present work focuses on identifying higher vertical impacts that are likely to be bone protective, classification of accelerometer outputs according to impacts may also be important for other health outcomes. For example, a close relationship is likely to exist between higher vertical impacts and lower limb muscle function, which we are planning to explore further based on contemporaneous jumping mechanography data collected in COSHIBA and MAC. As well as classification of PA according to impact level, the raw accelerometry trace provides opportunities to extract other important PA characteristics, such as duration of specific activities [27].

### Methodological considerations

We applied a new method to provide the first description of day-to-day levels of PA in older people based on classification of accelerometer recordings according to level of vertical impacts, including those above 1.5 g which may have bone protective properties. We included four distinct cohorts which provided a relatively large sample size of over 1500 participants with which to describe habitual impacts in older adults and included variation in terms of physical function, age, gender and health status. In particular, we were able to contrast findings from three population-based cohorts, with MAC which was recruited on the basis of high levels of PA. Our method of measuring high impacts seemed feasible in that the majority of participants who agreed to participate found it acceptable, and of the 1612 who wore the monitor, 94 % had usable data and valid recordings were obtained for an average of five out of a possible 7 days.

Although accelerometry is useful in providing an objective measure of PA, this method has a number of inherent limitations. For example, an individual’s daily activity may be altered as a result of being recorded. In addition, PA levels are likely to be affected by seasonal influences, although data collection was avoided during prolonged periods of ice and snow which would have significantly restricted outdoors activity. On further exploration, there was little evidence of seasonal differences in accelerometry counts within each of the three bands. For example, median counts for COSHIBA in band 3 were 35 in winter, 51 in spring, 45 in summer and 33 in autumn (one-way ANOVA of log accelerometry counts in each season: *p* = 0.31). In the absence of a smoothing algorithm, our method for recognising acceleration peaks related to individual movements, based on identification of accelerations higher than readings immediately before and after, could conceivably over-estimate these. However, we previously observed good agreement between number of impacts during an aerobics class as measured by our automated system as compared with manual analysis [16]. Although our sampling frequency of 50 Hz was limited due to battery life, this should have been sufficient to detect each individual movement, but may have underestimated peak accelerations if these occurred either side of the sampling time-point. The population-based cohort participants are unlikely to be entirely representative of the general older adult population, since they are already longstanding members of a scientific cohort, and had chosen to participate in an additional study investigating PA patterns. The demographic data indicated that most VIBE participants were of a high social class and in good physical health, and that NSHD, COSHIBA and HCS participants were of lower BMI and greater educational attainment compared with other cohort members, providing further evidence of selection. However, to the extent that our findings may have limited generalisability, if anything the true picture in terms of day-to-day level of high-impact exposure in older individuals is likely to be even lower than that reported here.

## Conclusions

We found that population-based older adults perform very limited amounts of PA producing higher vertical impacts as defined using the 1.5-g threshold. This observation was based on a new accelerometry-based method, which appears to provide valid measures of high vertical impacts, based on comparison of activity levels between different cohorts, and with PA levels as assessed by questionnaire. Further studies are justified to examine the benefits of high impacts defined in this way for skeletal health, for example by analysing these data in relation to bone measures collected concurrently in COSHIBA and MAC.

## Electronic supplementary material

ESM 1 (DOCX 39 kb)
