# Supplementary material for: A novel accelerometer-based method to describe day-to-day exposure to potentially osteogenic vertical impacts in older adults: findings from a multi-cohort study
Source: Osteoporos Int. 2016 Oct 31;28(3):1001–11. doi: 10.1007/s00198-016-3810-5 (PMC5306163; doi:10.1007/s00198-016-3810-5)
Supplement: Supplementary file 1 — (DOCX 39 kb) [file 198_2016_3810_MOESM1_ESM.docx]

**Supplementary material**

**Osteoporosis International**

# A novel accelerometer-based method to describe day-to-day exposure to potentially osteogenic vertical impacts in older adults: findings from a multi-cohort study

*Hannam K^1^, Deere KC^1^, Hartley A^1^, Clark EM^1^, Coulson J^2^, Ireland A^2^, Moss C^3^, Edwards MH^3^, Dennison E^3^, Gaysin T^4^, Cooper R^4^, Wong A^4^, McPhee JS^2^, Cooper C^3^, Kuh D^4^, Tobias JH^1^*

*^1^Musculoskeletal Research Unit, University of Bristol School of Clinical Sciences*

*^2^School of Healthcare Sciences, Manchester Metropolitan University*

*^3^MRC Lifecourse Epidemiology Unit, University of Southampton*

*^4^MRC Unit for Lifelong Health and Ageing at UCL*

**Corresponding author:-**

Professor Jon Tobias,

Musculoskeletal Research Unit, University of Bristol School of Clinical Sciences, Southmead Hospital, Bristol BS10 5NB, UK

Jon.Tobias@bristol.ac.uk

**Supplementary table 1: Categorisation of self-reported activities carried out in past 7 days according to level of impact**

| **Non-impact** | **Low-impact** | **Moderate-impact** | **High-impact** |
| --- | --- | --- | --- |
| cycling | aqua aerobics | aerobics | sprinting |
| light gardening | bowls | badminton |  |
| swimming | heavy gardening | dancing |  |
| water sports | golf | football/hockey |  |
|  | gym | running/jogging |  |
|  | hiking | squash |  |
|  | housework | tennis |  |
|  | snow skiing |  |  |
|  | tai chi/yoga/pilates |  |  |
|  | walking |  |  |
|  | manual handling |  |  |
|  | weights/heavy throwing |  |  |

**Supplementary table 2: Approximate self-reported hours spent in moderate-high impact activities by cohort**

|  | **NSHD** | *total n=686* | **COSHIBA** | *total n=449* | **MAC** | *total n=259* | **HCS** | *total n=118* | **Total** | *total n=1 512* |
| --- | --- | --- | --- | --- | --- | --- | --- | --- | --- | --- |
|  | *n (%)* | *mean (SD)* | *n (%)* | *mean (SD)* | *n (%)* | *mean (SD)* | *n (%)* | *mean (SD)* | *n (%)* | *mean (SD)* |
| **Aerobics** | 22 (3.20) | 1.91 (4.26) | 49 (10.91) | 2 (0.79) | 28 (10.81) | 2 (0.94) | 2 (1.69) | 2 (0) | 101 (6.68) | 1.98 (0.76) |
| **Badminton** | 16 (2.33) | 2.31 (0.79) | 5 (1.11) | 2.6 (0.89) | 9 (3.47) | 1.33 (0.5) | 0 | 0 | 30 (1.98) | 2.07 (0.87) |
| **Dancing** | 36 (5.25) | 2.17 (0.81) | 53 (11.80) | 2.36 (0.98) | 15 (5.79) | 2.07 (1.10) | 7 (5.93) | 2.71 (1.11) | 111 (7.34) | 2.28 (0.96) |
| **Football/hockey** | 1 (0.15) | 2 (0) | 2 (0.45) | 2.5 (2.12) | 9 (3.47) | 1.44 (0.53) | 0 | 0 | 12 (0.79) | 1.67 (0.89) |
| **Running/jogging** | 17 (2.48) | 1.65 (0.93) | 3 (0.67) | 2 (1) | 236 (91.12) | 3.37 (0.92) | 2 (1.69) | 3.5 (0.71) | 258 (17.06) | 3.24 (1.02) |
| **Squash** | 3 (0.44) | 2 (0) | 0 | 0 | 7 (2.70) | 1.14 (0.38) | 0 | 0 | 10 (0.66) | 1.4 (0.52) |
| **Tennis** | 11 (1.60) | 2.45 (0.82) | 4 (0.89) | 2.25 (0.5) | 8 (3.09) | 1.75 (0.89) | 0 | 0 | 23 (1.52) | 2.17 (0.83) |
| **Sprinting** | 0 | 0 | 0 | 0 | 4 (1.54) | 3 (1.54) | 0 | 0 | 4 (0.26) | 3 (1.54) |

Table shows the number and percentage of participants within each cohort who self-reported to participate in each activity categorised as moderate-high impact and the mean (standard deviation) of the approximate hours spent in that activity over a one week period.

**Supplementary table 3: Comparison of basic demographics between cohort members who did and did not participate in the accelerometry data collection of the VIBE study**

|  |  | **Completed accelerometry** | | **Invited- did not participate** | |  |
| --- | --- | --- | --- | --- | --- | --- |
| **Variable** | **Cohort** | *N* | *Mean (SD)* | *N* | *Mean (SD)* | *p diff* |
| **Age**† | NSHD | N/A |  | N/A |  |  |
|  | COSHIBA | 449 | 76.8 (3.1) | 615 | 79.3 (4.3) | <0.001 |
|  | HCS | 114 | 78.5 (2.6) | 294 | 78.6 (2.6) | 0.725 |
|  |  | **Completed accelerometry** | | **Remainder of the cohort** | |  |
|  |  | *N****** | *Mean (SD) / %* | *N** | *Mean (SD) / %* | *p diff* |
| **BMI** | NSHD^1^ | 626 | 27.2 (4.0) | 1242 | 28.2 (5.1) | <0.001 |
|  | COSHIBA^2^ | 401 | 26.4 (4.6) | 2384 | 27.1 (5.0) | 0.011 |
|  | HCS^3^ | 114 | 26.2 (4.0) | 2873 | 27.4 (4.4) | 0.003 |
| **Education level** |  |  |  |  |  |  |
| *None* | NSHD^1^ | 659 | 28.1 | 1366 | 32.7 | 0.004 |
| *O levels or equivalent* |  |  | 27.0 |  | 28.9 |  |
| *A levels of equivalent* |  |  | 30.4 |  | 28.6 |  |
| *Degree of higher* |  |  | 14.6 |  | 9.7 |  |
| *CSE or equivalent* | COSHIBA^2^ | 438 | 23.7 | 2651 | 40.8 | <0.001 |
| *Apprenticeship or equivalent* |  |  | 21.9 |  | 23.2 |  |
| *O level or equivalent* |  |  | 22.6 |  | 15.6 |  |
| *A level or equivalent* |  |  | 17.6 |  | 9.3 |  |
| *University degree or equivalent* |  |  | 10.3 |  | 4.4 |  |
| *Other* |  |  | 1.4 |  | 1.9 |  |
| *Don't know/can't remember* |  |  | 2.5 |  | 4.8 |  |
| *Age left full-time education* | HCS^3^ | 114 |  | 3110 |  | 0.291 |
| <=14 |  |  | 14.9 |  | 18.8 |  |
| >=15 |  |  | 85.1 |  | 81.2 |  |

† Age taken from start of each respective VIBE data collection time point (HCS: March 2014. COSHIBA: January 2015). Age not applicable to NSHD as the cohort were all born in the same week of the same year.

*****Maximum N with data on accelerometry in NSHD=686 and COSHIBA=449, but N varies due to missing data on covariates. Comparison group in NSHD is those who completed the home visit during which the invitation to participate in the VIBE accelerometry study was made, but who do not have accelerometry data.

^1^ BMI assessed at age 60-64 and educational level attained by age 26 in the NSHD

^2^ BMI and education data collected at COSHIBA baseline (2007-2009)

^3^ BMI and education data were collected at the HCS baseline (1998 – 2004)

| **Questionnaire variable** | **Low accelerometry band** | | | | | | **Medium accelerometry band** | | | | | | **Higher accelerometry band** | | | | | |
| --- | --- | --- | --- | --- | --- | --- | --- | --- | --- | --- | --- | --- | --- | --- | --- | --- | --- | --- |
|  | N | *Median* | *Beta* | *lower CI* | *Upper CI* | *p* | *N* | *Median* | *Beta* | *lower CI* | *Upper CI* | *p* | *N* | *Median* | *Beta* | *lower CI* | *Upper CI* | *p* |
|  |  |  |  |  |  |  |  |  |  |  |  |  |  |  |  |  |  |  |
| **Miles walked each day (since age 50)** | 413 | 8801.31 | 0.47 | 0.35 | 0.59 | <0.01 | 413 | 338.92 | 0.62 | 0.45 | 0.78 | <0.01 | 413 | 41.50 | 0.45 | 0.29 | 0.62 | <0.01 |
| *<1 mile* | 108 | 4389.57 |  |  |  |  | 108 | 146.25 |  |  |  |  | 108 | 24.79 |  |  |  |  |
| *1-2miles* | 207 | 9616.89 |  |  |  |  | 207 | 360.65 |  |  |  |  | 207 | 49.46 |  |  |  |  |
| *3-5miles* | 78 | 12481.47 |  |  |  |  | 78 | 489.28 |  |  |  |  | 78 | 58.42 |  |  |  |  |
| *>5miles* | 20 | 23526.10 |  |  |  |  | 20 | 1011.71 |  |  |  |  | 20 | 90.80 |  |  |  |  |
| **No. flight of stairs in typical day** | 434 | 8610.86 | 0.19 | 0.11 | 0.26 | <0.01 | 434 | 338.75 | 0.24 | 0.14 | 0.35 | <0.01 | 434 | 41.06 | 0.12 | 0.02 | 0.22 | 0.02 |
| *None* | 59 | 4514.21 |  |  |  |  | 59 | 109.52 |  |  |  |  | 59 | 25.29 |  |  |  |  |
| *1-2* | 23 | 6699.78 |  |  |  |  | 23 | 264.17 |  |  |  |  | 23 | 39.90 |  |  |  |  |
| *3-4* | 72 | 8254.44 |  |  |  |  | 72 | 283.90 |  |  |  |  | 72 | 35.14 |  |  |  |  |
| *5-10* | 169 | 9194.36 |  |  |  |  | 169 | 348.70 |  |  |  |  | 169 | 41.66 |  |  |  |  |
| *>10* | 111 | 11031.41 |  |  |  |  | 111 | 506.29 |  |  |  |  | 111 | 50.96 |  |  |  |  |
| **Self-reported walking speed** | 435 | 8608.78 | 0.57 | 0.48 | 0.66 | <0.01 | 435 | 338.59 | 0.69 | 0.56 | 0.83 | <0.01 | 435 | 41.25 | 0.43 | 0.29 | 0.57 | <0.01 |
| *Unable to walk* | 1 | 746.80 |  |  |  |  | 1 | 22.60 |  |  |  |  | 1 | 6.78 |  |  |  |  |
| *Very slow* | 36 | 1615.37 |  |  |  |  | 36 | 72.82 |  |  |  |  | 36 | 20.94 |  |  |  |  |
| *Stroll at easy pace* | 102 | 5577.02 |  |  |  |  | 102 | 170.52 |  |  |  |  | 102 | 30.91 |  |  |  |  |
| *Normal speed* | 192 | 9296.47 |  |  |  |  | 192 | 371.16 |  |  |  |  | 192 | 41.89 |  |  |  |  |
| *Fairly brisk* | 96 | 14771.92 |  |  |  |  | 96 | 667.03 |  |  |  |  | 96 | 69.57 |  |  |  |  |
| *Fast* | 8 | 25072.54 |  |  |  |  | 8 | 963.25 |  |  |  |  | 8 | 109.96 |  |  |  |  |
| **Activities in past 7 days (approx. hours)** |  |  |  |  |  |  |  |  |  |  |  |  |  |  |  |  |  |  |
| *non-impact* |  |  | 0.14 | 0.08 | 0.20 | <0.01 |  |  | 0.17 | 0.08 | 0.26 | <0.01 |  |  | 0.14 | 0.05 | 0.22 | <0.01 |
| *low-impact* |  |  | 0.09 | 0.07 | 0.12 | <0.01 |  |  | 0.12 | 0.08 | 0.16 | <0.01 |  |  | 0.09 | 0.06 | 0.13 | <0.01 |
| *moderate-high impact* |  |  | 0.14 | 0.06 | 0.22 | <0.01 |  |  | 0.21 | 0.09 | 0.32 | <0.01 |  |  | 0.23 | 0.12 | 0.33 | <0.01 |

**Supplementary table 4: Linear regression analyses exploring associations between accelerometry and physical activity questionnaire data in COSHIBA**

Table shows associations between number of low (0.5<g<1.0g), medium (1.0<g <1.5) and higher (≥1.5g) impacts normalised to 7 days and self-reported PA in COSHIBA. Median represents median number of accelerometry counts within the low, medium and high accelerometry band for each questionnaire category. Beta coefficients represent change in log accelerometry counts across PA questionnaire categories (and change in log accelerometry counts per hour of reported activity)

**Supplementary table 5: Linear regression analyses exploring associations between accelerometry and physical activity questionnaire data in HCS**

| **Questionnaire variable** | **Low accelerometry band** | | | | | | **Medium accelerometry band** | | | | | | **Higher accelerometry band** | | | | | |
| --- | --- | --- | --- | --- | --- | --- | --- | --- | --- | --- | --- | --- | --- | --- | --- | --- | --- | --- |
|  | N | *Median* | *Beta* | *lower CI* | *Upper CI* | *p* | *N* | *Median* | *Beta* | *lower CI* | *Upper CI* | *p* | *N* | *Median* | *Beta* | *lower CI* | *Upper CI* | *p* |
| **Miles walked each day (since age 50)** | 109 | 6406.82 | 0.42 | 0.13 | 0.70 | <0.01 | 109 | 213.26 | 0.29 | -0.06 | 0.64 | 0.11 | 109 | 39.99 | 0.15 | -0.19 | 0.49 | 0.39 |
| *<1 mile* | 28 | 2953.14 |  |  |  |  | 28 | 169.83 |  |  |  |  | 28 | 43.55 |  |  |  |  |
| *1-2miles* | 52 | 7154.10 |  |  |  |  | 52 | 265.96 |  |  |  |  | 52 | 35.33 |  |  |  |  |
| *3-5miles* | 20 | 6211.99 |  |  |  |  | 20 | 227.22 |  |  |  |  | 20 | 28.92 |  |  |  |  |
| *>5miles* | 9 | 12426.89 |  |  |  |  | 9 | 183.23 |  |  |  |  | 9 | 46.09 |  |  |  |  |
| **No. flight of stairs in typical day** | 112 | 6404.24 | 0.42 | 0.23 | 0.61 | <0.01 | 112 | 203.80 | 0.46 | 0.22 | 0.70 | <0.01 | 112 | 38.63 | 0.41 | 0.18 | 0.64 | <0.01 |
| *None* | 12 | 2122.10 |  |  |  |  | 12 | 54.82 |  |  |  |  | 12 | 16.85 |  |  |  |  |
| *1-2* | 10 | 2416.45 |  |  |  |  | 10 | 83.28 |  |  |  |  | 10 | 34.63 |  |  |  |  |
| *3-4* | 16 | 3395.60 |  |  |  |  | 16 | 116.69 |  |  |  |  | 16 | 21.12 |  |  |  |  |
| *5-10* | 48 | 7527.26 |  |  |  |  | 48 | 273.31 |  |  |  |  | 48 | 47.05 |  |  |  |  |
| *>10* | 26 | 10592.81 |  |  |  |  | 26 | 506.47 |  |  |  |  | 26 | 113.09 |  |  |  |  |
| **Self-reported walking speed** | 112 | 6404.24 | 0.77 | 0.55 | 1.00 | <0.01 | 112 | 203.80 | 0.91 | 0.63 | 1.20 | <0.01 | 112 | 38.63 | 0.54 | 0.23 | 0.84 | <0.01 |
| *Unable to walk* | 0 | 0.00 |  |  |  |  | 0 | 0.00 |  |  |  |  | 0 | 0.00 |  |  |  |  |
| *Very slow* | 11 | 1463.31 |  |  |  |  | 11 | 43.00 |  |  |  |  | 11 | 12.73 |  |  |  |  |
| *Stroll at easy pace* | 39 | 3578.55 |  |  |  |  | 39 | 92.10 |  |  |  |  | 39 | 28.61 |  |  |  |  |
| *Normal speed* | 47 | 8857.03 |  |  |  |  | 47 | 304.96 |  |  |  |  | 47 | 58.38 |  |  |  |  |
| *Fairly brisk* | 10 | 20439.74 |  |  |  |  | 10 | 1392.53 |  |  |  |  | 10 | 126.27 |  |  |  |  |
| *Fast* | 5 | 8176.43 |  |  |  |  | 5 | 566.10 |  |  |  |  | 5 | 45.21 |  |  |  |  |
| **Activities in past 7 days (approx hours)** |  |  |  |  |  |  |  |  |  |  |  |  |  |  |  |  |  |  |
| *non-impact* |  |  | 0.07 | -0.10 | 0.25 | 0.42 |  |  | 0.05 | -0.17 | 0.27 | 0.67 |  |  | 0.01 | -0.20 | 0.23 | 0.91 |
| *low-impact* |  |  | 0.15 | 0.10 | 0.21 | <0.01 |  |  | 0.16 | 0.08 | 0.23 | <0.01 |  |  | 0.09 | 0.01 | 0.16 | 0.02 |
| *moderate-high impact* |  |  | 0.13 | -0.14 | 0.39 | 0.35 |  |  | 0.30 | -0.02 | 0.62 | 0.07 |  |  | 0.43 | 0.12 | 0.73 | 0.01 |

Table shows associations between number of low (0.5<g<1.0g), medium (1.0<g <1.5) and higher (≥1.5g) impacts normalised to 7 days and self-reported PA in HCS. Median represents median number of accelerometry counts within the low, medium and high accelerometry band for each questionnaire category. Beta coefficients represent change in log accelerometry counts across PA questionnaire categories (and change in log accelerometry counts per hour of reported activity).

**Supplementary table 6: Linear regression analyses exploring associations between accelerometry and physical activity questionnaire data in NSHD**

| **Questionnaire variable** | **Low accelerometry band** | | | | | | **Medium accelerometry band** | | | | | | **Higher accelerometry band** | | | | | |
| --- | --- | --- | --- | --- | --- | --- | --- | --- | --- | --- | --- | --- | --- | --- | --- | --- | --- | --- |
|  | N | *Median* | *Beta* | *lower CI* | *Upper CI* | *p* | *N* | *Median* | *Beta* | *lower CI* | *Upper CI* | *p* | *N* | *Median* | *Beta* | *lower CI* | *Upper CI* | *p* |
| **Miles walked each day (since age 50)** | 639 | 14771.34 | 0.30 | 0.21 | 0.39 | <0.01 | 639 | 801.19 | 0.34 | 0.21 | 0.47 | <0.01 | 639 | 90.32 | 0.16 | 0.43 | 0.00 | <0.01 |
| *<1 mile* | 153 | 9641.88 |  |  |  |  | 153 | 411.16 |  |  |  |  | 153 | 57.40 |  |  |  |  |
| *1-2miles* | 274 | 15748.33 |  |  |  |  | 274 | 874.71 |  |  |  |  | 274 | 96.20 |  |  |  |  |
| *3-5miles* | 157 | 19492.54 |  |  |  |  | 157 | 1204.64 |  |  |  |  | 157 | 103.46 |  |  |  |  |
| *>5miles* | 55 | 21506.77 |  |  |  |  | 55 | 882.98 |  |  |  |  | 55 | 96.36 |  |  |  |  |
| **No. flight of stairs in typical day** | 652 | 14611.90 | 0.10 | 0.04 | 0.16 | <0.01 | 652 | 800.82 | 0.16 | 0.07 | 0.25 | <0.01 | 652 | 91.72 | 0.06 | 0.24 | 0.00 | <0.01 |
| *none* | 89 | 10932.67 |  |  |  |  | 89 | 569.54 |  |  |  |  | 89 | 62.93 |  |  |  |  |
| *1-2* | 39 | 17286.19 |  |  |  |  | 39 | 990.57 |  |  |  |  | 39 | 96.13 |  |  |  |  |
| *3-4* | 78 | 12227.23 |  |  |  |  | 78 | 540.64 |  |  |  |  | 78 | 59.28 |  |  |  |  |
| *5-10* | 262 | 14593.26 |  |  |  |  | 262 | 822.85 |  |  |  |  | 262 | 95.27 |  |  |  |  |
| *>10* | 184 | 15911.23 |  |  |  |  | 184 | 959.52 |  |  |  |  | 184 | 127.45 |  |  |  |  |
| **Self-reported walking speed** | 652 | 14567.75 | 0.51 | 0.42 | 0.61 | <0.01 | 652 | 800.82 | 0.67 | 0.53 | 0.81 | <0.01 | 652 | 91.72 | 0.44 | 0.73 | 0.00 | <0.01 |
| *Unable to walk* | 0 | 0.00 |  |  |  |  | 0 | 0.00 |  |  |  |  | 0 | 0.00 |  |  |  |  |
| *Very slow* | 16 | 2526.43 |  |  |  |  | 16 | 113.10 |  |  |  |  | 16 | 21.62 |  |  |  |  |
| *Stroll at easy pace* | 92 | 8503.80 |  |  |  |  | 92 | 346.85 |  |  |  |  | 92 | 55.50 |  |  |  |  |
| *Normal speed* | 343 | 13338.20 |  |  |  |  | 343 | 751.48 |  |  |  |  | 343 | 83.42 |  |  |  |  |
| *Fairly brisk* | 183 | 21026.38 |  |  |  |  | 183 | 1479.70 |  |  |  |  | 183 | 139.26 |  |  |  |  |
| *Fast* | 18 | 26636.44 |  |  |  |  | 18 | 1559.51 |  |  |  |  | 18 | 215.98 |  |  |  |  |
| **Activities in past 7 days (approx hours)** |  |  |  |  |  |  |  |  |  |  |  |  |  |  |  |  |  |  |
| *non-impact* |  |  | 0.07 | 0.03 | 0.11 | <0.01 |  |  | 0.09 | 0.03 | 0.15 | 0.01 |  |  | 0.09 | 0.06 | 0.13 | <0.01 |
| *low-impact* |  |  | 0.07 | 0.05 | 0.09 | <0.01 |  |  | 0.10 | 0.07 | 0.13 | <0.01 |  |  | 0.08 | 0.05 | 0.11 | <0.01 |
| *moderate-high impact* |  |  | 0.17 | 0.08 | 0.25 | <0.01 |  |  | 0.21 | 0.08 | 0.34 | <0.01 |  |  | 0.34 | 0.22 | 0.47 | <0.01 |

Table shows associations between number low (0.5<g<1.0g), medium (1.0<g <1.5) and higher (≥1.5g) impacts normalised to 7 days and self-reported PA in the NSHD. Median represents median number of accelerometry counts within the low, medium and high accelerometry band for each questionnaire category. Beta coefficients represent change in log accelerometry counts across PA questionnaire categories (and change in log accelerometry counts per hour of reported activity).
